# Estimating the burden of dengue and the impact of release of wMel *Wolbachia*-infected mosquitoes in Indonesia: a modelling study

**DOI:** 10.1186/s12916-019-1396-4

**Published:** 2019-09-09

**Authors:** Kathleen M. O’Reilly, Emilie Hendrickx, Dinar D. Kharisma, Nandyan N. Wilastonegoro, Lauren B. Carrington, Iqbal R. F. Elyazar, Adam J. Kucharski, Rachel Lowe, Stefan Flasche, David M. Pigott, Robert C. Reiner, W. John Edmunds, Simon I. Hay, Laith Yakob, Donald S. Shepard, Oliver J. Brady

**Affiliations:** 1 Department of Disease Control, Faculty of Infectious Tropical Diseases, London School of Hygiene & Tropical Medicine, London, UK; 2 Centre for Mathematical Modelling of Infectious Diseases, London School of Hygiene & Tropical Medicine, London, UK; 3 Department of Infectious Disease Epidemiology, Faculty of Epidemiology and Public Health, London School of Hygiene & Tropical Medicine, London, UK; 4 Heller School for Social Policy and Management, Brandeis University, Waltham, MA USA; 5 Faculty of Medicine, Public Health and Nursing, Universitas Gadjah Mada, Yogyakarta, Indonesia; 6 Oxford University Clinical Research Unit, Wellcome Trust Asia-Africa Programme, Ho Chi Minh City, Vietnam; 7 Nuffield Department of Medicine, University of Oxford, Oxford, UK; 8 Eijkman Oxford Clinical Research Unit, Eijkman Institute for Molecular Biology, Jakarta, Indonesia; 9 Department of Health Metrics Sciences, Institute for Health Metrics and Evaluation, University of Washington, Seattle, WA USA

**Keywords:** Dengue, Burden, *Wolbachia*, Elimination, Maps, Model, Indonesia

## Abstract

**Background:**

*Wolbachia*-infected mosquitoes reduce dengue virus transmission, and city-wide releases in Yogyakarta city, Indonesia, are showing promising entomological results. Accurate estimates of the burden of dengue, its spatial distribution and the potential impact of *Wolbachia* are critical in guiding funder and government decisions on its future wider use.

**Methods:**

Here, we combine multiple modelling methods for burden estimation to predict national case burden disaggregated by severity and map the distribution of burden across the country using three separate data sources. An ensemble of transmission models then predicts the estimated reduction in dengue transmission following a nationwide roll-out of wMel *Wolbachia*.

**Results:**

We estimate that 7.8 million (95% uncertainty interval [UI] 1.8–17.7 million) symptomatic dengue cases occurred in Indonesia in 2015 and were associated with 332,865 (UI 94,175–754,203) lost disability-adjusted life years (DALYs). The majority of dengue’s burden was due to non-severe cases that did not seek treatment or were challenging to diagnose in outpatient settings leading to substantial underreporting. Estimated burden was highly concentrated in a small number of large cities with 90% of dengue cases occurring in 15.3% of land area. Implementing a nationwide *Wolbachia* population replacement programme was estimated to avert 86.2% (UI 36.2–99.9%) of cases over a long-term average.

**Conclusions:**

These results suggest interventions targeted to the highest burden cities can have a disproportionate impact on dengue burden. Area-wide interventions, such as *Wolbachia*, that are deployed based on the area covered could protect people more efficiently than individual-based interventions, such as vaccines, in such dense environments.

**Electronic supplementary material:**

The online version of this article (10.1186/s12916-019-1396-4) contains supplementary material, which is available to authorized users.

## Background

Dengue is a mosquito-borne viral disease that has one of the world’s fastest growing burden [1]. Despite substantial investments, existing vector control methods, such as insecticides, have proved insufficient to sustainably control dengue [2]. Novel arbovirus vector control tools are needed, and a range of alternative approaches are currently under development to meet this need [3, 4]. Mosquitoes infected with *Wolbachia*, a naturally occurring bacterium, experience reduced rates of dengue virus (DENV) infection, and female mosquitoes can pass the bacterium on to the next generation, allowing *Wolbachia*-infected mosquitoes to replace the wild-type population [5]. Release of male mosquitoes infected with *Wolbachia* can also be used for population suppression due to inviable mating with female *wild-type* mosquitoes. Early releases of mosquitoes infected with the wMel *Wolbachia* strain have shown promising replacement results, and suppression strategies with other strains are currently being tested in different countries around the world [6–9].

An added advantage of a population replacement strategy is that *Wolbachia* reduces replication of other arboviruses within the mosquito, including chikungunya, yellow fever and Zika viruses [10, 11], and potentially offers the better longer-term strategy. Given such replacement programmes are self-sustaining, investment in a well-coordinated and properly monitored release campaign over 2 to 3 years could have many years of benefit. Existing releases at the local and city level have proven that *Wolbachia*-infected mosquitoes can replace the wild-type *Aedes aegypti* population and persist for at least 7 years’ post-release [12]. Epidemiological evidence of effectiveness is also growing, and a cluster randomised controlled trial is currently underway in the city of Yogyakarta [13]. The next phase of development for *Wolbachia* will be to expand from single-site operations to coordinated sub-national roll-out.

As the most populous country in dengue-endemic South East Asia, Indonesia is consistently estimated to be among the three countries with the largest dengue burden [14–16]. However, due to high rates of asymptomatic infection and symptoms which are not easily distinguishable from many other infections, the number of dengue cases is still highly uncertain. Accurate, contemporary estimates of the burden of dengue in Indonesia are needed to quantify the benefits of any scale-up in DENV control. Fully detailing how the economic and case burden of dengue is distributed over space, by disease severity and financial responsibility can help inform investment in new control tools. This is particularly important for diseases such as dengue where the burden is dominated by morbidity rather than mortality [15]. Milder dengue cases are nearly always underreported [17], and the costs of illness by various parties often hidden [18]. When combined with model-based estimates of the impact of the intervention, burden estimates can be used to map where new interventions, such as *Wolbachia*, are likely to have the biggest effect and can be used for evaluating eventual impact.

A major challenge to understanding the impact of interventions against DENV is an accurate estimation of baseline disease burden. Estimates of disease burden for specific settings are often scarce due to limited availability of data on the sub-clinical community-based burden of dengue including asymptomatic and mildly symptomatic cases. Efforts to estimate the burden of dengue can be categorised into either a bottom-up approach, where the primary focus is to estimate the total number of cases through community-based surveys for infection [14], then divide into different levels of severity, or top-down approach where reported case numbers are multiplied by “expansion factors” to correct for underreporting [16]. Multiple previous studies have estimated the burden of dengue in Indonesia [14–16, 19–21] using a variety of data sources and methods, but it is difficult to assess consensus among them due to the differences in data sources, methods, case definitions and assumptions about transmission.

Three types of data are typically available for mapping the spatial distribution of dengue burden: occurrence (presence/absence), case incidence and seroprevalence (lifetime prevalence). Seroprevalence data contain the most information about long-term average burden in a given location, but few such surveys have been conducted, typically resulting in less information about the geographic variation. Occurrence data, on the other hand, is geographically ubiquitous, but many other factors determine how the presence of a disease translates into case numbers. Existing approaches to map dengue risk have been dominated by ecological niche modelling using occurrence data [22–24] with a focus on mapping the distribution rather than the burden of dengue. Maps of reported dengue incidence at increasingly high spatial resolution are routinely used by ministries of health but are rarely combined with models to account for variations over time, reporting biases and quantification of uncertainty. Some attempts have been made to map seroprevalence data directly in areas with sufficient surveys [25]. However, these contrasting approaches have never formally been compared to identify their strengths and weaknesses for mapping burden. There is also a lack of consensus on how useful extrapolating from data in other countries or transmission settings is for mapping burden in any one given country.

In the current absence of cluster randomised control trial results for *Wolbachia*, estimates of effectiveness have been obtained by combining vector competence studies with mathematical models of DENV transmission [26]. A range of DENV transmission models have been published and, despite some fundamental differences in their structures, consensus results about the effects of interventions can be drawn [27]. Even with the imperfect reduction of DENV dissemination in the mosquito, substantial reductions in population-level burden can be achieved, even in very high-transmission settings [26, 28, 29]. However, the critical relationship between baseline transmission intensity and *Wolbachia* effectiveness is yet to be demonstrated in the field. Further, how control might be impacted by the highly heterogeneous transmission intensities routinely observed across small spatial scales [30–32] remains unknown. It is possible that if the impact on transmission is small, this may just increase the average age of secondary, typically more severe, DENV infection to older more vulnerable age groups; thus a detailed consideration of DENV immunology is needed in such assessments.

Here, we produce the most up-to-date, detailed and robust estimates of the burden of dengue in Indonesia; map burden at a high spatial resolution throughout the country; and predict the effect of a widespread *Wolbachia* programme in different locations.

## Methods

### Estimating national burden and breakdown by setting

#### Case burden

**Fig. 1:**
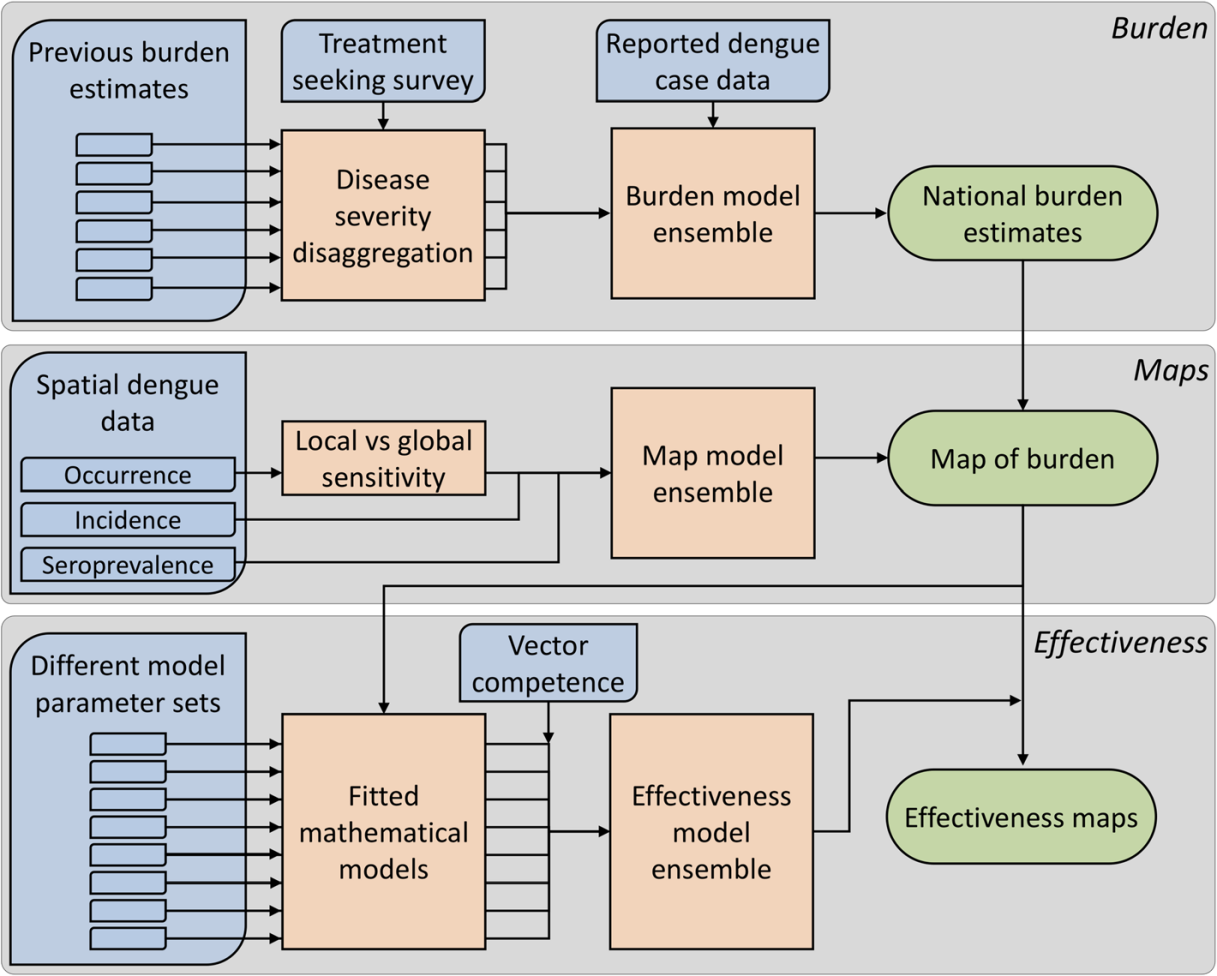
Schematic overview of the methods. Blue boxes indicate data, orange boxes modelling/analysis and green boxes outputs

Multiple previous studies have estimated the burden of dengue in Indonesia [14–16, 19–21] using a variety of different data sources and independent methods, and use case definitions that vary in disease severity. In this analysis, we standardise (i) the case definitions across existing estimates, (ii) the reference year and (iii) the denominator population size for each estimate. We then produce an ensemble estimate for the total burden disaggregated by disease severity (Fig. 1).

We estimate burden at four levels of severity, with each DENV infection resulting in one of these four, mutually exclusive final outcomes:

1. *Self-managed* cases disrupt the routine of the individual (e.g. not going to work or school) but do not result in seeking treatment at a formal private or public healthcare facility. Such cases may be untreated, self-treated (e.g. using medicines from a pharmacy) or treated in informal settings.
2. *Outpatient* cases are severe enough for formal medical treatment to be sought but are managed on an outpatient-basis, e.g. dengue (ambulatory) clinics.
3. *Hospitalised* cases are severe enough to require hospital admission and repeated observation by trained medical staff.
4. *Fatal* cases whereby acute DENV infection is the leading cause of death.

For burden estimation methods that were missing estimates of burden at any of these levels of severity, new estimates were created using our own rates of care seeking and hospitalisation. Care-seeking rates were obtained from a nationally representative survey (SUSENAS [33]) that asked about treatment seeking for fever which was assumed to be representative for dengue (Additional file 1: SI1.1.). Hospitalisation rates were taken from the control arm results of a recent dengue vaccine trial in Indonesia [19] adjusted for age (Additional file 1: SI1.2, Table S2).

The final breakdown of symptomatic cases is shown in Additional file 1: Table S1. All burden estimation methods that produced estimates of absolute “symptomatic” cases, i.e. disease at any level of severity, were apportioned into their sub-categories using the values in Additional file 1: Table S1. For the expansion factor-based methods [19–21] (i.e. those that predicted the ratio of true number of cases per one case reported), we multiplied the expansion factor by the annual average number of cases reported by the Indonesian Ministry of Health (national branch) between 2014 and 2016 (*n* = 144,736, to derive an estimate for the reference year of 2015). These reported cases represent a mix of clinical and laboratory-confirmed (NS1 antigen of IgM/IgG positive) cases in line with the SEARO-WHO case definition [34], with a small subset tested using molecular methods (PCR) to estimate regional serotype composition. To standardise absolute burden estimates to this reference year, we proportionally adjusted the estimates based on population change over this time period using UN population estimates [35]. The posterior distribution of the consensus estimate was simulated using a simple ensemble approach where 1000 random samples were drawn from lognormal or normal distributions parameterised using the mean and 2.5–97.5% uncertainty intervals [UIs] of each of the burden estimates (with equal weighting between studies, Additional file 1: Table S4).

#### DALYs

DALY estimates for hospitalised and non-hospitalised cases were obtained from Zeng et al [36] Years of life lost were calculated from the age-stratified case data using life expectancies based on Indonesia health statistics [37] and were not discounted.

### Mapping the spatial distribution of dengue burden

#### Mapping data

Three different datasets on occurrence, incidence and seroprevalence of dengue were used to estimate the spatial variation in dengue cases. Our updated dengue occurrence database [10.6084/m9.figshare.8243168] includes 626, 3701 and 13,604 unique point and polygon locations where dengue has previously been reported in Indonesia, South East Asia and globally, respectively (Additional file 1: Table S5). A corresponding database of 330, 681 and 9039 locations where Japanese encephalitis, West Nile fever, Zika and chikungunya have been reported were used as background points for national, South East Asia and global analyses, respectively. These diseases share similar clinical, epidemiological or diagnostic features to dengue, and we assume that the occurrence of these diseases is indicative of the ability to diagnose and report arboviral diseases including dengue. We therefore assume a report of these diseases is indicative of an absence of dengue at that particular time and place. Incidence was obtained from the aforementioned official data disaggregated into 333 regencies and cities (admin 2 areas).

Age-stratified seroprevalence studies (age range 1–18) have recently been conducted across 30 admin 2 areas in 2014 [38, 39] which were used to estimate the long-term average force of infection using simple catalytic models fitted with a binomial likelihood [25] (Additional file 2).

#### Mapping covariates

All mapping models contained covariates for (i) gross domestic product (using a demographic downscaling method described in [40]), (ii) annual cumulative precipitation (from the intergovernmental panel on climate change general circulation model projections [41]), (iii) minimum annual relative humidity (using a temperature-based dewpoint calculator [40, 42]), (iv) mosquito suitability for *Ae. aegypti* and *Ae. albopictus* [43], (v) urban/rural status [40] and (vi) temperature suitability for DENV transmission [44] all at a 5 × 5 km resolution for the year 2015 [45]. For data points representative at the admin 2 level (incidence, seroprevalence data and selected polygon occurrence data), population-weighted averages of each covariate were calculated over their corresponding region.

#### Mapping models

Three distinct mapping models fit relationships between the above covariates and the three different measures risk: (i) occurrence, (ii) incidence and (ii) force of infection calculated from seroprevalence. Within each model, 100 bootstrapped generalised boosted regression models (GBMs) were fit to capture data uncertainty. For the presence/absence occurrence data, boosted regression trees (BRT) with a binary Bernoulli distribution were fitted [40, 46], while incidence and force of infection models were fit with Poisson distributed GBMs (see Additional file 1: SI1.3. for parameter settings and code [10.6084/m9.figshare.8243168]). A sensitivity analysis was also performed to assess the occurrence data model sensitivity to local, regional and global data (Additional file 1: SI1.3.). Simpler generalised linear models with automated variable selection were also fit for incidence and seroprevalence data to assess the relative prediction improvements with more complex model structures (Additional file 1: SI1.3.).

The risk maps created by each of these mapping models was multiplied by a population surface [47] then standardised to the estimated national burden total from the ensemble of burden models. This assumed a linear correlation between mapped risk and burden [14, 48]. A posterior distribution of predicted incidence for each 5 × 5 km pixel was derived from an ensemble of each three burden maps with the probability of sampling inversely proportional to the within mapping model variance among the 100 sub-BRT models.

### Introduction of a *Wolbachia* programme to reduce dengue

#### Mathematical modelling

A human age-structured deterministic dynamic mathematical model of DENV infection was used to determine the impact of a wMel *Wolbachia* programme in Indonesia (Additional file 1: SI1.4.). Individuals were assumed to be born susceptible and upon exposure will develop primary DENV infection. We assumed that upon recovery, an individual will go through a period of temporary cross-immunity, and afterwards, the individual is assumed to only be susceptible to heterologous serotypes. Serotype-specific exposure is not modelled explicitly, but sequential reductions in susceptibility due to homologous immunity and a maximum of four lifetime infections allow the model to replicate multi-serotype behaviour assuming all four serotypes are omnipresent (Additional file 1: SI1.4.). All individuals that develop infection were assumed to be equally infectious, and this was independent of disease severity [49]. We do not explicitly account for DENV infection within mosquitoes but assume that human-mosquito-human transmission is accounted for within the transmission coefficient. For each stage of infection, the probability of being symptomatic, hospitalised or fatal was assumed to vary based on the different model parameterisations from a previous dengue modelling comparison exercise Flasche et al. [27] (Additional file 1: Table S6–S7). To capture the uncertainty in these values, eight sub-models were created with identical structure but different parameters for disease severity, duration of infectiousness and duration of temporary cross-immunity.

#### Fitting the mathematical model to burden estimates

The model transmission coefficient was estimated by fitting (using least squares) to unique values of symptomatic incidence as predicted by our burden and mapping analyses for each of the eight model parameterisations. Symptomatic cases was chosen as a fitting metric because the variation would closely align with variation in the transmission rate, as opposed to variation in assumed hospitalisation rates that vary across models. The best-fitting transmission coefficient values were obtained using a rejection MCMC algorithm with a 5% tolerance on the symptomatic case incidence rates. Our analysis aimed to quantify long-term average estimates of transmission then predict the effectiveness with the disease at equilibrium. However, dengue in Indonesia, as of 2015, is not currently at equilibrium. Continual, urban nationwide transmission of dengue has only been present in Indonesia from circa 1988 onwards [50], meaning there is currently a higher proportion of susceptible individuals and thus higher incidence rates than there will be once the disease reaches long-term equilibrium. To enable our model to fit these temporarily high symptomatic case incidence rates, we reduced the life expectancy to 27 (2015–1988) years by imposing 100% mortality after the 27th year to represent the shorter period of exposure during transmission coefficient fitting. For high reported incidence where model estimates are outside of the 5% tolerance, the nearest fitting parameter estimate was selected as we assumed that these high incidence values were representative of anomalous years or symptomatic case rates. This only affected < 3% of values but may underestimate transmission and thus overestimate *Wolbachia* effectiveness in very high-transmission environments. After obtaining accurate estimates of the transmission parameter, it was applied to a model with current-day realistic Indonesian life expectancy and age distribution (Additional file 1: Figure S1). The ability of this model to reconstruct accurate age-specific seroprevalence was assessed (Additional file 1: Figure S2), then it was used to simulate symptomatic case incidence with and without *Wolbachia* to calculate the effectiveness at equilibrium.

#### Vector competence reduction

The clinical and field entomological data of vector competence of wMel-infected *Ae. aegypti* in Carrington et al. [51] were used to estimate the reduction in transmission associated with a *Wolbachia* programme. A logistic regression model of the extrinsic incubation period (EIP) in mosquitoes was fitted to observe the reduced rate at which DENV disseminates from the ingestion of a blood meal to the presence in the mosquito salivary glands in *Wolbachia*-infected compared to wild-type mosquitoes (Additional file 1: SI1.5, Figure S3, Additional file 1: Figure S4). Separate models fit for each serotype and high- and low-viremia blood meals which were assumed representative of hospitalised and non-hospitalised cases, respectively.

#### Incorporating the impact of a *Wolbachia* programme

Estimates of the reduction in vectorial capacity in *Wolbachia*-infected mosquitoes (Additional file 1: SI1.5) were used to proportionally reduce transmission coefficients in the DENV transmission model which was then run until endemic equilibrium was reached (100 years) with an average life expectancy of 65 years, consistent with the Indonesian population age distribution (Additional file 1: Figure S1). The impact of the *Wolbachia* programme is estimated as 1- (symptomatic incidence post-*Wolbachia*/symptomatic incidence pre-*Wolbachia*). For each model parameterisation, this gave point estimates of efficacy for a range of different values of baseline transmission intensity (as measured by incidence of hospitalised cases). To create a smooth, continually decreasing function between these two variables, monotonically decreasing thin-plate splines were fit using the “scam” package in R (Additional file 1: Figure S7). Simulation from a normal distribution defined by the mean and standard error of the fit of the spline model was used to build a distribution of effectiveness values for each DENV model parameterisation (eight parameterisations). An ensemble prediction of effectiveness was then derived by the sum of predictions from the individual models (equal weighting). This relationship was then applied to each map pixel with 1000 realisations of burden and effectiveness to build up a predicted distribution of burden before and after release of *Wolbachia*-infected mosquitoes. All code used in these analyses is available from the following repository [10.6084/m9.figshare.8243168].

## Results

### Case burden of dengue by disease severity

**Fig. 2:**
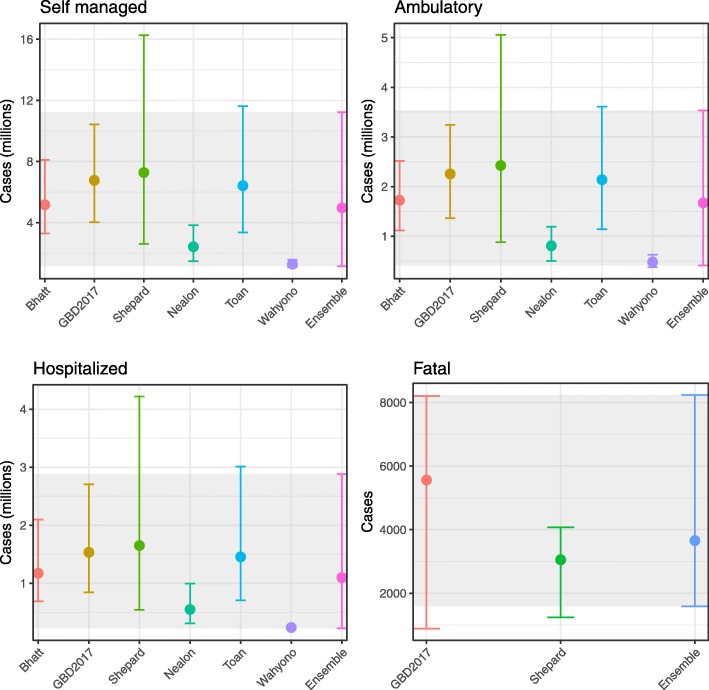
Previous estimates for the burden of dengue in Indonesia adjusted for the year of 2015 (colours) and our ensemble estimate (grey shading) at different levels of disease severity

To obtain consensus estimates of the burden of dengue in Indonesia, we take a simple unweighted ensemble of multiple previous approaches (Fig. 2). We found that nearly all previous burden estimates had overlapping credible intervals with Bhatt et al., GBD2017; Shepard et al.; and Toan et al. estimates having the closest concordance [1, 14, 16, 20]. The estimate by Wahyono et al. [21], which was the only method to estimate underreporting solely using Delphi panel interviews of dengue experts, was consistently lower than all other estimates for all disease severities and underrepresented the degree of uncertainty relative to other estimation methods. Our combined ensemble captured uncertainty in both the individual models and uncertainty about model choice and is thus broad, particularly at lower disease severity levels.

We estimate that 7.8 million (UI 1.8–17.7 million) symptomatic dengue cases occurred in Indonesia in the reference year 2015 (average 2014–2016) or approximately 1 in 31 people (Table 1). Among these, we estimate 64% were self-managed with over the counter medicines or other forms of informal healthcare. A further 22% were seen as outpatients with limited opportunity for diagnosis of dengue and were never admitted. Despite this large proportion of non-hospitalised dengue, we still predict that 1.1 million (0.22–2.9) hospitalised dengue cases occurred in Indonesia in 2015, among which 3658 (1590–8240) died, equating to a hospitalised case fatality rate of 0.33% (0.29–0.71). Only 100,347, 129,689 and 204,172 dengue cases (mostly hospitalised) were reported to the ministry of health in the years of 2014, 2015 and 2016, respectively. Assuming only hospitalised cases are reported, this would suggest only 12% (UI 7–45%) of hospitalised cases are reported.

**Table 1:** The total estimated burden of dengue in Indonesia in 2015 by case severity and disability-adjusted life years (DALYs)

Outcome	Absolute number in thousands (95% UI)	Percentage share (95% UI)
Fatal	3.658 (1.59–8.24)	0.05 (0.05–0.09)
Hospitalised	1102 (224–2883)	14.20 (12.63–16.33)
Outpatient	1675 (409–3535)	21.59 (20.02–23.00)
Self-managed	4977 (1142-11,233)	64.16 (63.61–64.28)
Total	7757 (1778-17,660)	100
YLDs	245 (56–556)	73.6 (59.5–73.7)
YLLs	88 (38–198)	26.4 (26.3–40.5)
DALYs	333 (94–753)	100

95% uncertainty intervals (UI) are shown for all predictions. UIs for percentage share are based on the mean totals

*YLD* years lost to disability, *YLL* years of life lost

By combining these case estimates with the reported age distribution of dengue cases in Indonesia and severity-specific disability weights [36], we estimate a total of 332,865 (UI 94,175–754,203) DALYs are lost due to dengue each year in Indonesia of which 73.6% are due to disability and 26.4% due to fatality (Table 1). This further emphasises the contribution of non-fatal and non-severe outcomes to dengue burden.

### Mapping dengue burden

#### Comparing local to global data for producing national risk maps

As occurrence data was available globally, we first performed a sensitivity analysis to the geographic scope of data. Using data just from Indonesia will maximise representativeness of local DENV epidemiology but may fail to capture the full range of environmental space in which dengue can be transmitted in the country. The opposite is true of using global datasets. We find that using a regional dataset from across South East Asia offers the best compromise between accurately predicting occurrence data from Indonesia (mean area under the curve [AUC] 0.95) while still maintaining a good multivariate environmental coverage (mean Multivariate Environmental Similarity Score [MESS] > 0 for 88% of Indonesian land area, Additional file 1: Figure S5).

#### Comparing occurrence, incidence and seroprevalence data for mapping burden

**Fig. 3:**
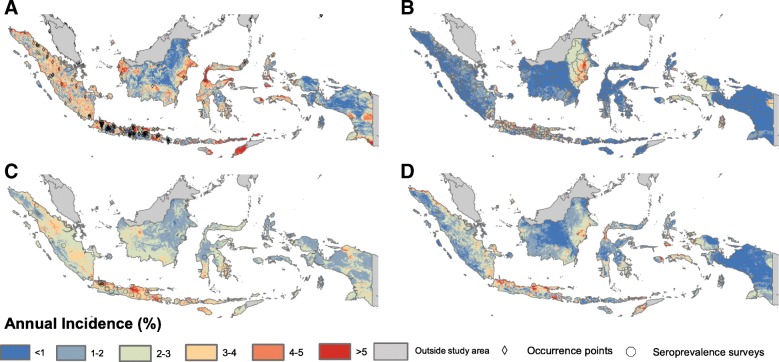
The spatial distribution of annual incidence of symptomatic dengue cases in Indonesia as predicted by models fit to the **a** occurrence data **b** reported case data, **c** seroprevalence data and **d** the mean of an ensemble of each data type. The spatial location of the data points and polygons for each map are also shown. Pearson correlation coefficients between pixels are as follows: **a**, **b** 0.15, **a**–**c** 0.24 and **b**, **c** 0.15 (all non-significant). The full map ensemble (not just the mean) is used for all subsequent analyses

We found that dengue risk maps fitted to occurrence, incidence and seroprevalence datasets gave contrasting risk maps with some areas of consensus. While more complex GBM model structures gave a better fit for incidence data (*R*^2^ 0.171 vs 0.022, Additional file 1: Table S10), simpler generalised linear models (GLMs) explained more variance within the smaller seroprevalence dataset (*R*^2^ 0.112 vs 0.082, Additional file 1: Table S10). All maps agreed that the highly populated urban regions of Java, West Kalimantan and Northern Sumatra conferred higher risk. The map using reported case data (Fig. 3b) tended to predict lower incidence in more remote areas than the other two maps (e.g. Sulawesi and Timor). Generally, maps based on seroprevalence data (Fig. 3c) predicted little geographic heterogeneity; maps based on reported cases (Fig. 3b) estimated high geographic concentration in particular areas with maps based on occurrence (Fig. 3a) somewhere between the two. Given the strengths and limitations of each of these different data sources, our final map consisted of an ensemble of each of these three maps weighted by their relative bootstrap predictive variance. While the ensemble propagated the uncertainty around the distribution of dengue through the rest of the analysis, a mean map of the ensemble is given in Fig. 3d.

### Spatial concentration of dengue burden

**Fig. 4:**
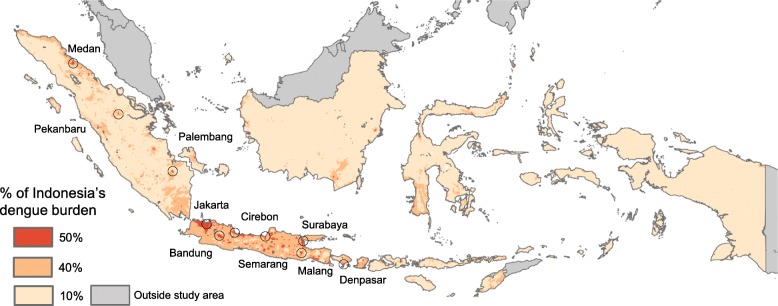
Predicted spatial concentration in dengue burden. The minimum spatial area that contains 50% (red) then 40% (orange) of dengue burden. The 10 cities with the highest predicted burden are also shown

Because our maps suggest dengue is ubiquitous throughout Indonesia, the urbanised nature of the population in Indonesia ensures that the case burden of dengue is highly spatially concentrated. Fifty per cent of the 7.8 million cases are concentrated in just 1.08% of the land area and 90% of cases in just 15.26%. This spatial concentration of burden presents a key advantage for control strategies with costs that scale with the area (as opposed to the number of people) such as *Wolbachia* (Fig. 4).

In Indonesia, 14.7% of total dengue burden is concentrated in just ten cities that together make up only 0.35% of the land area (Table 2). These cities do, however, also make up 15.0% of the national population, implying that the concentration of dengue burden is due to the highly urbanised distribution of Indonesia’s population. This makes dengue a good candidate for targeted interventions, particularly for interventions that focus on immobile vector populations.

**Table 2:** Top 10 cities in Indonesia with the highest estimated dengue burden

City	Predicted cases (all severities, thousands, 95% UI)	Percentage of national burden (95% UI)	Cumulative percentage of national burden	Cumulative percentage of national population	Cumulative percentage of national area
1. Jakarta*	515.2 (108–1439)	7.7 (6.3–9.5)	7.7	8.8	0.14
2. Kota Bandung	79.8 (17–222)	1.2 (1.0–1.5)	8.9	9.9	0.15
3. Surabaya	73.9 (18–231)	1.2 (1.0–1.3)	10.1	11.0	0.16
4. Medan	66.8 (15–189)	1.0 (0.9–1.1)	11.1	11.8	0.18
5. Semarang	54.3 (12–143)	0.8 (0.6–1.0)	11.9	12.4	0.20
6. Cirebon	47.3 (10–120)	0.7 (0.6–0.8)	12.6	13.1	0.25
7. Pekanbaru	39.8 (9–112)	0.6 (0.5–0.7)	13.2	13.5	0.31
8. Palembang	38.6 (8–100)	0.6 (0.4–0.7)	13.8	14.1	0.32
9. Kota Malang	30.7 (7–85)	0.5 (0.3–0.6)	14.3	14.5	0.33
10. Denpasar	29.6 (5–87)	0.4 (0.3–0.7)	14.7	15.0	0.35

*City of Jakarta includes the satellite cities of Bekasi, Tangerang, South Tangerang, Depok and Bogor

### Predicted reduction in dengue burden achievable through a *Wolbachia* programme

Predicting the potential reduction in dengue burden achievable by a nationwide *Wolbachia* programme requires considering several stages in the transmission process.

Our re-analysis of the vector competence data from [51] combined with mosquito survival rates suggested an average 56% (95% confidence interval [CI] 54–58%) reduction in the probability of onward transmission from a mosquito infected from a non-severe (low viremia) dengue case (Additional file 1: Table S8). This percentage reduction was slightly higher for DENV4 (60%, CI 59–62) and considerably lower for severe (high viremia) cases (47–50% for DENV1–3, 54% for DENV4).

**Fig. 5:**
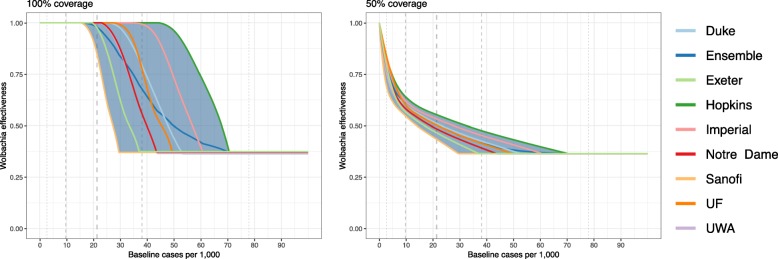
Reductions in hospitalised dengue cases at equilibrium after the introduction of *Wolbachia* as predicted by a mathematical model using eight different parameterisations from previously published models. Baseline incidence is the number of hospitalised dengue cases per million before the introduction of *Wolbachia*. Ensemble mean and 95% uncertainty intervals are shown in dark blue. One hundred per cent coverage forms the baseline scenario for subsequent analyses. Vertical dotted lines show the 1, 25, 50, 75 and 99th percentiles of the estimated symptomatic incidence in areas across Indonesia

To assess what impact these reductions in transmission would have on case burden at different transmission intensities, we used an ensemble of mathematical models with eight different parameterisations (Fig. 5). There was a consensus among the models that *Wolbachia* could achieve elimination in low transmission settings (baseline incidence of symptomatic cases < 5 per thousand). Models also agreed on a gradual decrease in effectiveness (% reduction in cases after *Wolbachia* introduced) as transmission intensity increased, albeit at considerably different rates (Fig. 5, Additional file 1: Figure S7). Models with parameterisations based on the DENV models from Sanofi predicted the lowest effectiveness of *Wolbachia* while those from Hopkins predicted the highest effectiveness (Fig. 5).

**Fig. 6:**
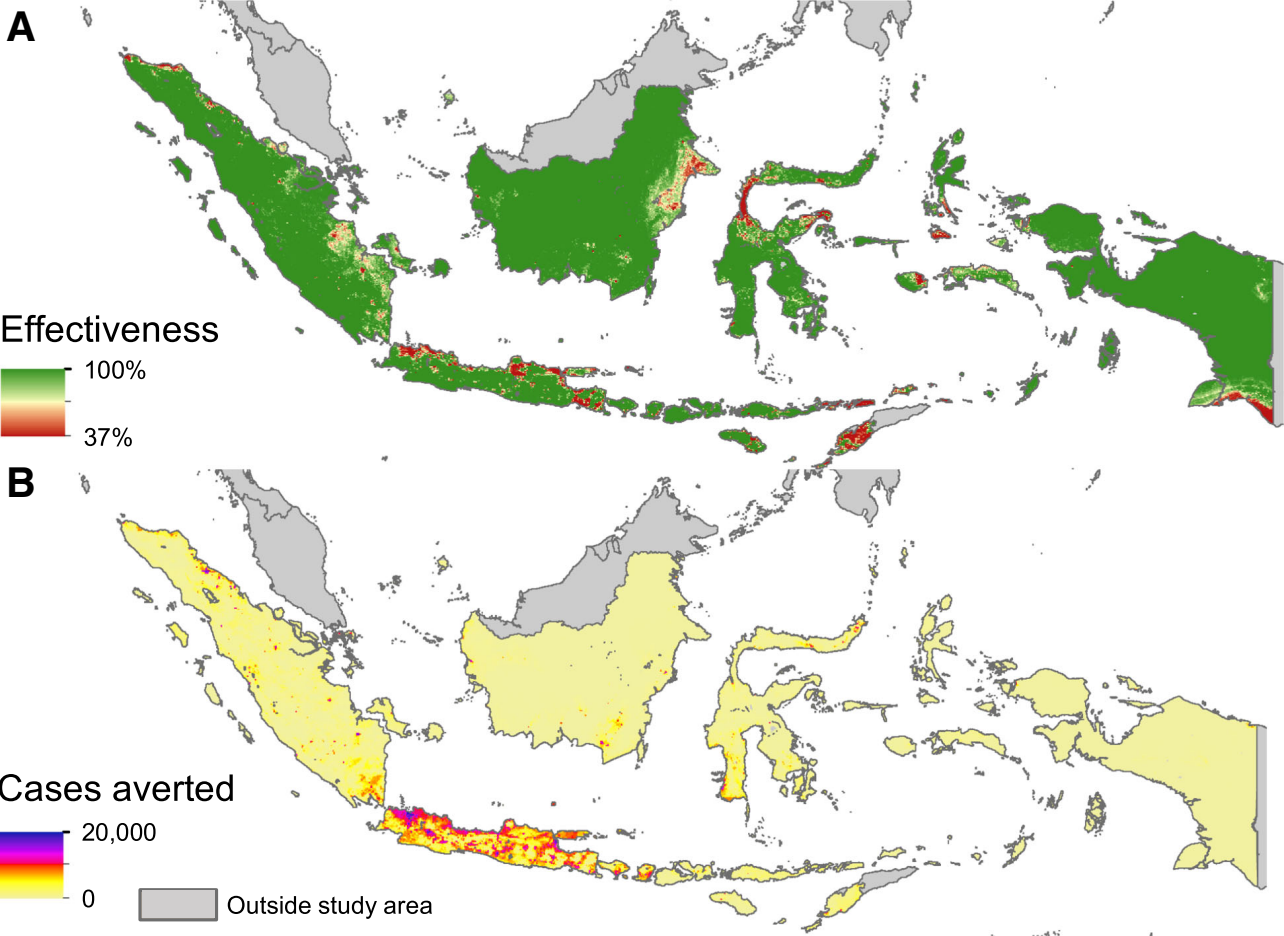
Maps of effectiveness (**a**) and averted symptomatic cases per year (**b**) from a nationwide homogeneous *Wolbachia* programme with 100% coverage

Finally, applying these effectiveness functions to the maps and burden estimates allowed us to map the effectiveness and symptomatic cases averted across Indonesia (Fig. 6). This showed that while effectiveness is lower in the high transmission intensity cities (Fig. 6a), if *Wolbachia* can be deployed in each area for approximately equivalent cost, the number of cases averted (and thus cost-effectiveness) will be higher in urban areas (Fig. 6b).

Overall, we predict that a national roll-out of *Wolbachia* at 100% coverage could achieve a long-term average of 86.2% (UI 36.2–99.9%) reduction in cases of all severities, potentially averting 6.7 million symptomatic cases, 947 thousand hospitalisations and 3154 deaths a year based on 2015 burden figures (Table 3).

**Table 3:** Predicted annual number of cases of dengue averted by a nationwide release of *Wolbachia*-infected mosquitoes

Self-managed	Outpatient	Hospitalised	Fatal	Total	DALYs	Percentage reduction
4,290,379 (413,657–11,163,893)	1,442,623 (147,587–3,567,030)	946,971 (81,545–2,909,260)	3154 (569–8118)	6,683,127 (643,358–17,648,301)	290,002 (38,604–727,567)	86.2% (36.2–99.9%)

Numbers in brackets are 95% uncertainty intervals

## Discussion

In this paper, we produce comprehensive estimates of the burden of dengue in Indonesia and find that a large proportion of cases self-manage their own disease (64%, 5.0 million) or are treated in outpatient departments (22%, 1.7 million). We use multiple mapping methods and data sources to show that the spatial distribution of dengue risk is heterogeneous even in an endemic country such as Indonesia. The highly urbanised nature of the population means that 14.7% of the national burden is concentrated in just 10 cities. Finally, we show that a nationwide *Wolbachia* campaign could (over the long term) avert a significant proportion of burden (86.2%, UI 36.2–99.9%) with elimination predicted in low transmission settings.

The high spatial concentration of dengue burden in cities, in highly urbanised countries such as Indonesia, presents opportunities for targeted control strategies. In particular, *Wolbachia*, which is deployed on a per-km^2^ basis, could offer major scaling advantages over vaccines, which are deployed on a per-person basis, in areas with high population density. The large number of people covered by a focal *Wolbachia* programme has the potential to outweigh the reduced efficacy of the intervention in these high transmission settings, and formal cost-effectiveness analysis is needed to compare the investment cases between urban and rural areas.

This work adds to a growing body of evidence that the majority of the burden of dengue is attributable to morbidity rather than mortality [14, 15, 19, 52]. The large number of self-limiting mild infections contributes more to DALY burden than the small number of infections that result in severe or fatal manifestations. Many of these mild cases do not seek treatment, are not clinically diagnosable and thus do not have any opportunity to be reported in routine health statistics. These results can be used to assess the hidden economic burden of the disease and to estimate the cost-effectiveness of interventions for dengue [16, 27]. Our results also suggest that only 12% (UI 7–45%) of hospitalised cases are reported. While lower than the regional average (42%) [17], underreporting of dengue is not unusual and may occur for a variety of reasons including lack of reporting in the private sector, misdiagnosis and limited coverage of the surveillance system [53].

A key limitation of our analysis is the wide uncertainty intervals for our final estimates of burden, and thus predicted efficacy of *Wolbachia*. This arises due to the limited quantity and variable quality of datasets detailing the treatment-seeking behaviour for dengue [17], reliability of diagnosis and underreporting of identified cases. In this study, we chose to ensemble different burden estimation methods with equal weighting due to different data sources and methodological approaches challenging any formal assessment of quality or comparativeness. Initiatives such as the WHO burden estimation toolkit [53] aim to provide guidance to countries on how to conduct burden estimation for dengue and aim to generate more standardised and internationally comparable data for dengue burden estimation. Additionally, while using the national SUSENAS survey to estimate the treatment-seeking rates was a great strength due to its sample size and comprehensive design, it did require assuming that treatment seeking for fever is comparable to treatment seeking for dengue. As fever is one of the milder symptoms of dengue [54], this may have underestimated rates of seeking care [55].

Different data sources suggest different spatial distributions of dengue risk. This is partly because each data source has strengths and weaknesses for measuring different aspects of dengue’s distribution (summarised in Additional file 1: Table S11) [23]. Occurrence data is most informative about the extent of transmission, incidence about temporal variation and seroprevalence about long-term risk of infection. Occurrence and incidence data may also be subject to spatial reporting bias, e.g. higher probability of reporting in urban areas, which may lead us to overestimate the concentration of risk in high-density areas. We tried to overcome this by using notifications of other infectious diseases (which are also subject to the same biassed sampling frame) as background points, and the relative influence statistics (Additional file 1: Table S9) and covariate effects plots (Additional file 1: Figure S6) do not suggest simple univariate drivers of dengue’s distribution in Indonesia. Disease mapping frameworks have been suggested that would enable simultaneous joint inference of the distribution and observation bias of multiple rare diseases and could improve occurrence maps for diseases that share similar characteristics but limited data [56]. Future work will attempt to more formally define relationships between occurrence, incidence and seroprevalence data and their relationship with burden to enable joint inference that accounts for the accuracies, sensitivities and biases in each data source [57].

Our mathematical model assumed a stable prevalence of *Wolbachia* in the wild *Aedes* population and only focussed on the long-term stable-state effectiveness. With the high levels of herd immunity currently present in Indonesia, it is possible that elimination would temporarily be achieved even in high transmission intensity areas and short-term impact would generally likely be higher than predicted here [58]. Our analysis of vector competence data only compared dissemination rates to the mosquito salivary glands in lab-reared (not-field caught) mosquitoes. Effectiveness may be higher in the field due to the effect field conditions impose on the mosquito immune system and the availability of nutritional resources [51]. Due to the lack of available vector competence data, we were only able to model the reduction in transmission due to one strain of *Wolbachia* (wMel) and one vector species (*Ae. aegypti*). *Ae. albopictus*, a known secondary DENV vector, is also present in Indonesia, although it typically has a more rural distribution and its role in sustaining dengue transmission in this setting remains unclear [59]. Different *Wolbachia* strains also vary in their DENV-blocking dynamics, their effects on mosquito longevity and can be affected by local conditions, e.g. temperature [60], meaning further reductions in DENV transmission may be possible. Finally, our modelling comparison exercise only used the parameter estimates from each of the models, not the model structures themselves, which may include additional uncertainty and provide further insights into the effectiveness of *Wolbachia* and its variation across transmission intensity. Our current estimates are in agreement with earlier work suggesting elimination is achievable in low transmission intensity but not high transmission intensity environments [26]. This raises the possibility that *Wolbachia* may need to be combined with a range of other dengue control tools in high endemicity environments. The key strength of this analysis is that it is the most detailed analysis of Indonesia’s dengue burden to date. We combine multiple modelling and mapping approaches with multiple datasets and fully propagate uncertainty at each step through to our final results.

Future work will include pairing these burden estimates and impact predictions with economic data on the costs of dengue illness and of deploying *Wolbachia* in different areas. This will allow estimates of the cost-effectiveness of *Wolbachia* programmes and estimates of how it varies throughout Indonesia that can be used to quantify the costs and benefits of future investments in wide-scale releases and inform different release strategies.

## Conclusion

In this paper, we use various mathematical modelling approaches to estimate the current burden of dengue in Indonesia. We estimate a total of 7.8 million (UI 1.8–17.7 million) symptomatic cases occurred in 2015 with a high proportion not seeking treatment and not being reported to the national surveillance system. Despite this, the concentration of disease burden in large cities offers hope of targeted dengue control. Releasing *Wolbachia*-infected mosquitoes is one option that we predict could ultimately avert over three quarters of the country’s current disease burden. Past experience with dengue interventions [27] has taught us to take an optimistic but cautious, conservative and diverse approach to such projections that considers all potential routes of failure and their subsequent impact on cost-effectiveness. However, given early evidence of epidemiological effectiveness [7] and a general desire to see *Wolbachia* scaled up, model-based projections have an important role to play in advising decision-makers on maximising the impact.

## Additional files

Additional file 1: All supplementary methods and results. (DOCX 1194 kb)

Additional file 2: Force of infection estimates from each seroprevalence survey. (XLSX 1014 kb)

## Data Availability

All data and code used in the analyses are freely available from the following weblink: 10.6084/m9.figshare.8243168.

## Abbreviations

AUC: Area under the curve
BRT: Boosted regression trees
CI: Confidence interval
DALYs: Disability-adjusted life years
DENV: Dengue virus
EIP: Extrinsic incubation period
GBD2017: Global Burden of Disease Project 2017
GBM: Generalised boosted regression models
GLM: Generalised linear model
MESS: Multivariate Environmental Similarity Score
SEARO: South East Asian Regional Office
SUSENAS: Indonesian National Socioeconomic Survey
UI: Uncertainty interval
WHO: World Health Organization
YLD: Years lost to disease
YLL: Years of life lost
