# Supplementary material for: Estimating the burden of dengue and the impact of release of wMel Wolbachia-infected mosquitoes in Indonesia: a modelling study
Source: BMC Med. 2019 Sep 9;17:172. doi: 10.1186/s12916-019-1396-4 (PMC6732838; doi:10.1186/s12916-019-1396-4)
Supplement: Supplementary file 1 — All supplementary methods and results. (DOCX 1194 kb) [file 12916_2019_1396_MOESM1_ESM.docx]

*Table S1. Breakdown of symptomatic cases*

| All symptomatic dengue cases | % Sought no formal treatment | % Sought formal treatment | % Sought formal treatment AND were hospitalised |
| --- | --- | --- | --- |
| 100 | 64.1 (63.7 – 64.6) | 35.9 (35.4 – 36.6) | 14.5 (13.5 – 16.5) |

*Table S2: Age treatment seeking rates for fever as measured by the 2014 SUSENAS survey disaggregated by age. Percentages in brackets indicate 95% confidence intervals.*

|  | Age group | | | | |
| --- | --- | --- | --- | --- | --- |
|  | 0-4 | 5-14 | 15+ | 0-14 | 5+ |
| % people with fever who sought treatment formal healthcare treatment | 42.5  (41.9-43.2) | 38.1  (37.7-38.5) | 33.6  (33.4-33.9) | 40.3  (39.8-40.8) | 35.9  (35.4-36.3) |

*Table S3. Previous burden estimates included in our ensemble estimate, EF = Expansion Factor, BRT = Boosted Regression Trees, DHS = Demographic and Health survey, CFR = Case Fatality Rate, GDP = Gross Domestic Product*

| Burden estimate lead author | Data description | Methods description | Reference year of burden estimate | Original estimate definition | Post-adjustment estimate definitions |
| --- | --- | --- | --- | --- | --- |
| Bhatt | Dengue occurrence data for mapping, serological cohort studies for incidence estimates | BRT-based risk map is used to extrapolate incidence estimates globally | 2010 | Symptomatic* | Self-managed  Clinical outpatient  Clinical inpatient |
| GBD collaborators | vital registration, verbal autopsy, and dengue surveillance data combined with meta-analysis of EF studies | Cause of Death Ensemble modelling tool to estimate dengue fatalities, Expansion factor regression to estimate national case burden | 2016 | Symptomatic*  Fatal | Self-managed  Clinical outpatient  Clinical inpatient  Fatal |
| Shepard | GBD 2013 results, DHS data, CFR data, EF studies for inpatient / outpatient proportions | Population and GDP-based regression | 2013 | Symptomatic*  Fatal | Self-managed  Clinical outpatient  Clinical inpatient  Fatal |
| Nealon | Fever surveillance cohort  Passive surveillance data | Expansion factor calculation | 2011 | EF (reported : symptomatic) | Self-managed  Clinical outpatient  Clinical inpatient |
| Toan | Serological surveillance cohorts  Passive surveillance data | Expansion factor calculation | 2000-2002, 2010 | EF (reported : symptomatic) | Self-managed  Clinical outpatient  Clinical inpatient |
| Wahyono | Estimates from Delphi panel survey of dengue experts  Passive surveillance data | Expansion factor calculation for different types of dengue cases | 2015 | EF (reported : symptomatic)  EF (reported : outpatient)  EF (reported : inpatient) | Self-managed  Clinical outpatient  Clinical inpatient |

* symptomatic = disease of all levels of severity

SI 1.1. Treatment seeking rates

Because most existing burden estimates only estimate all symptomatic or all clinical dengue cases, additional data was included to divide these estimates among the above four outcomes. The National Socio-Economic Household survey (SUSENAS) is a quarterly survey of approximately 75,000 nationally-representative households across Indonesia that (until 2014) included questions on healthcare seeking and recent health events. We use annual data from the 2014 SUSENAS [1] to inform the number of symptomatic dengue cases that seek treatment at formal health facilities. We confine our analysis to individuals aged 5+ to be comparable with the age of reported dengue cases. We assume that treatment seeking behaviour for fever is representative of treatment seeking for dengue as fever is one of the most common symptoms of dengue and is one of the earliest symptoms to be observed in infection. Among all individuals aged 5+ who had reported a fever in the past month 35.9% (95% uncertainty interval [UI] 35.4-36.3%, Table S2) sought formal outpatient treatment, defined here as a hospital, a puskesmas (public community health clinic) or a private doctor/clinic.

SI 1.2. Hospitalisation rates

To derive the proportion of individuals who were hospitalised (among those who sought treatment), we used data from the control arm of the Indonesian site of the recent Sanofi Pasteur vaccine trial [2] which collected data on fever episodes due to dengue and hospitalisation among a cohort of 623 children in three cities in Indonesia. This gave a crude hospitalisation rate of 45.5% (dengue patients admitted to hospital / dengue patients that sought treatment). We applied an age adjustment to this rate to reflect suspected higher hospitalisation rates in children. We made an assumption that higher treatment seeking rates for children with fever were directly proportional to higher rates of hospital admission for dengue and use the relative rates from the SUSENAS survey to make this adjustment because it is the most plausible data source available for this adjustment. This relative rate between individuals aged 5+ (age of dengue patients in Indonesia) and individuals within the vaccine trial (aged 0-14) was 35.9 / 40.3 = 0.89 (Table S2). Applying this figure to the crude rate of hospitalisation for dengue gave an age-adjusted hospitalisation rate of 40.5%. Because uncertainty exists around this age-adjustment we included upper and lower bounds for this estimate. The upper bound corresponds to the non-age adjusted hospitalisation rate (45.5%), while the lower bound corresponds to an age adjustment that assumes all hospitalised dengue cases occur in individuals 15+ (45.5% * (33.6 / 40.3) = 38.2%). The rate of hospitalisation amongst dengue patients who sought treatment at an official healthcare facility was therefore estimated at 40.5% (38.2% - 45.5%).

*Table S4 Assigning distributions to the previous burden estimates. As the full distribution of uncertainty from each previous burden estimate was unavailable, we assigned either a normal or a log-normal distribution to each estimate based on fit to the stated means and 95% uncertainty intervals [UI] in the original estimates. All fitted distributions had means equally to the stated means and UIs as close as possible to the stated UIs as determined by least squares.*

| Previous burden estimate lead author | Dengue severity | % difference between stated 2.5% UI and distribution 2.5% UI | % difference between stated 97.5% UI and distribution 97.5% UI | Fitted distribution type |
| --- | --- | --- | --- | --- |
| Bhatt | symptomatic | -0.3174494 | -0.048351 | LogNormal |
| GBD2017 | symptomatic | -7.9582547 | -1.2620386 | LogNormal |
| Shepard | symptomatic | -24.148636 | -0.759593 | LogNormal |
| Nealon | symptomatic | -3.4766259 | -0.5341412 | LogNormal |
| Toan | symptomatic | -5.0948261 | -0.4452219 | LogNormal |
| Wahyono | symptomatic | 1.70650479 | 0.79195149 | LogNormal |
| Bhatt | clinical | -0.6979295 | -0.1153746 | LogNormal |
| GBD2017 | clinical | -8.4650369 | -1.2389551 | LogNormal |
| Shepard | clinical | -24.742544 | -0.7276411 | LogNormal |
| Nealon | clinical | -3.9145091 | -0.5575174 | LogNormal |
| Toan | clinical | -5.5564106 | -0.4525361 | LogNormal |
| Wahyono | clinical | 1.44163794 | 0.61676958 | LogNormal |
| Bhatt | ambulatory | -4.9030085 | -1.0141766 | LogNormal |
| GBD2017 | ambulatory | 3.21544739 | 1.35365258 | Normal |
| Shepard | ambulatory | -30.512925 | -1.1949148 | LogNormal |
| Nealon | ambulatory | -8.1752934 | -1.5340256 | LogNormal |
| Toan | ambulatory | -10.221561 | -1.1178712 | LogNormal |
| Wahyono | ambulatory | 1.00789188 | 0.36090927 | LogNormal |
| Bhatt | hospitalised | 4.42824241 | 0.45326237 | LogNormal |
| GBD2017 | hospitalised | -3.2814424 | -0.3345286 | LogNormal |
| Shepard | hospitalised | -18.149201 | -0.3616421 | LogNormal |
| Nealon | hospitalised | 1.2719077 | 0.11844973 | LogNormal |
| Toan | hospitalised | -0.060905 | -0.0037717 | LogNormal |
| Wahyono | hospitalised | 1.14638259 | 0.74926805 | LogNormal |
| GBD2017 | fatal | -114.944 | -12.420042 | Normal |

SI1.3 GBM model settings and sensitivity analysis

The choice of approach and starting values of parameters were chosen in-line with Elith et al. 2008 [3].

First, the dataset for each model was divided into a training, validation and testing set. To generate the training and validation datasets, data records were sampled with replacement until the original number of records was obtained. The validation (for independent or external validation of the model) dataset was then comprised of records not included in this sample. The combined training and testing dataset was then split 75% training 25% validation (bagging fraction of 0.75). This data partitioning procedure was repeated 100 times to generate 100 different models and validation statistics to average predictive performance over different data selection subsets.

The predictive performance of each model ensemble was assessed using the area under the curve [AUC] statistic in the testing dataset with pair-wise distance sampling [4]. To assess if the range of environmental and ecological conditions present in Indonesia were adequately covered by the training data (and thus the model), we also measured the percent of land area in Indonesia with Multivariate Environmental Similarity Score [MESS] greater than 0 [5,6]. MESS scores are calculated by comparing environmental conditions in the prediction area (all of Indonesia) to the environmental conditions covered by the data points. MESS scores are negative if at least one variable has a value outside the range of the original data points (see Supplement 3 in [5]for full details). Finally, fit of incidence and seroprevalence models was assessed by R^2^ between the natural logarithm of model predictions and the testing dataset.

Models were fit with an interaction depth of four to represent potentially important high dimensional interactions between covariates. The optimum learning rate (contribution of each regression tree to the overall model) and total number of each trees in each model were evaluated through their performance on the validation dataset (selected by minimum deviance). This is done in 10-fold step-wise cross-validation procedure [3] with initial values of the learning rate of 0.005 and the maximum number of trees set to 10000. The selected number of optimal trees did not exceed this initial specified value in any of the fitted models. All models were carried out using the “gbm” package in R (version 3.3.3) [7].

A further sensitivity analysis was performed for the occurrence data models to test the most appropriate scale of data to use. The following models were compared: i) a national model fitted to unique presence points from Indonesia, ii) a regional model fitted to unique presence points from all South East Asian countries plus Papua New Guinea and iii) a global model fitted to 19,992 unique presence points.

Finally, to test whether additional model complexity was justified we also fit simpler generalised linear models (GLMs) to incidence and seroprevalence datasets and compared their predictive ability on the test dataset against comparable predictions from the GBM models. These GLMs also included automated covariate selection by Akaike’s Information Criterion using the R package “glmulti” [8].

*Table S5: Number and types of data that were used in the geographic sensitivity analysis*

|  | National | Regional | Global |
| --- | --- | --- | --- |
| Point data | 361 | 1,937 | 8,552 |
| Polygon data | 265 | 1,764 | 5,052 |
| Background data | 330 | 681 | 9,039 |
| Total | 956 | 4,382 | 22,643 |

SI1.4. Detailed description of the mathematical model of dengue transmission.

The mathematical model is described using a set of differential equations. The equations describe an age-structured 4-stage model for dengue infection where individuals progress through 4 distinct Susceptible-Infected-Recovered stages of dengue while progressing through the yearly age classes (denoted by $(a)$ where the first age category has a distinct set of equations to account for births). The force of infection for each stage accounts for individuals being refractory to the number of dengue infections they have already experienced. Essentially, the force of infection of the second stage is approximately 0.75 of the value of the first stage, and so on;

$$\lambda_{1}(a)=1-{[1-S_{1}\left( a \right)\left( {\beta_{l}(I}_{1}\left( a \right)+I_{3}\left( a \right)+I_{4}\left( a \right) \right)+\beta_{h}I_{2}\left( a \right))]}^{4}$$

$$\lambda_{2}(a)=1-{[1-S_{2}\left( a \right)\left( {\beta_{l}(I}_{1}\left( a \right)+I_{3}\left( a \right)+I_{4}\left( a \right) \right)+\beta_{h}I_{2}\left( a \right))]}^{3}$$

$$\lambda_{3}(a)=1-{[1-S_{3}(a)\left( {\beta_{l}(I}_{1}\left( a \right)+I_{3}\left( a \right)+I_{4}\left( a \right) \right)+\beta_{h}I_{2}\left( a \right))]}^{2}$$

$$\lambda_{4}(a)=S_{4}(a)\left( {\beta_{l}(I}_{1}\left( a \right)+I_{3}\left( a \right)+I_{4}\left( a \right) \right)+\beta_{h}I_{2}\left( a \right))$$

The force of infection by stage is determined by the proportion of individuals infectious, the proportion of individuals susceptible (and previous infection), and the rate of infection, where primary, tertiary and quaternary rate of infection () is assumed to have a different rate to the rate of secondary infection (). In the absence of Wolbachia infected mosquitoes we assume that all stages of dengue infection are equally infectious (ie. $\beta_{h}=\beta_{l}$) which simplifies the equations for the force of infection. Consequently, only one parameter for transmission was estimated from the data. This assumption is consistent with 6 of the 8 models within Flasche et al., where the implications of this assumption are provided within Table S6. The age-distribution of the Indonesian population was modelled assuming a triangular distribution where up to a certain age (in this case 35 years) mortality was assumed to be minimal and after this period the proportion surviving to the next year was time-varying, resulting in a triangular shape until there were no individuals surviving after 80 years of age. Figure S1 illustrates the modelled age distribution against the reported age distribution from UN data. The following set of differential equations describe the transitions across stages and age-groups, consisting of 960 equations (80 age-groups with 12 infection classifications). The first age groups (S_1_(1), I_1_(1), R_1_(1), S_2_(1), etc.) are specified separately to older age groups as a (continuous) birth rate is included in S_1_(1) and there are no age-associated influxes for the remaining infection compartments (equivalent to $\phi\left( a-1 \right)=0$).


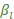

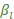

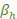

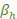

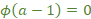

$$\frac{{dS}_{1}\left( 1 \right)}{dt}=b-\lambda_{1}(1)-(\phi(1)+\mu(1))S_{1}(1)$$

$$\frac{{dS}_{1}\left( a \right)}{dt}={\phi\left( a-1 \right)S}_{1}\left( a-1 \right)-\lambda_{1}\left( a \right)-{\left( \phi\left( a \right)+\mu\left( a \right) \right)S}_{1}\left( a \right)$$

$$\frac{{dI}_{1}\left( 1 \right)}{dt}=\lambda_{1}(1)-\gamma I_{1}(1)-{(\phi(1)+\mu(1))I}_{1}(1)$$

$$\frac{{dI}_{1}\left( a \right)}{dt}={\phi\left( a-1 \right)I}_{1}\left( a-1 \right)+\lambda_{1}\left( a \right)-\gamma I_{1}\left( a \right)-{\left( \phi\left( a \right)+\mu\left( a \right) \right)I}_{1}\left( a \right)$$

$$\frac{{dR}_{1}\left( 1 \right)}{dt}=\gamma I_{1}\left( 1 \right)-\omega R_{1}(1)-{(\phi(1)+\mu(1))R}_{1}(1)$$

$$\frac{dR_{1}\left( a \right)}{dt}={\phi\left( a-1 \right)R}_{1}\left( a-1 \right)+\gamma I_{1}\left( a \right)-\omega R_{1}\left( a \right)-{\left( \phi\left( a \right)+\mu\left( a \right) \right)R}_{1}\left( a \right)$$

$$\frac{{dS}_{2}\left( 1 \right)}{dt}=\omega R_{1}\left( 1 \right)-\lambda_{2}(1)-(\phi(1)+\mu(1))S_{2}(1)$$

$$\frac{{dS}_{2}\left( a \right)}{dt}={\phi\left( a-1 \right)S}_{1}\left( a-1 \right)+\omega R_{1}\left( a \right)-\lambda_{2}\left( a \right)-{\left( \phi\left( a \right)+\mu\left( a \right) \right)S}_{2}\left( a \right)$$

$$\frac{{dI}_{2}\left( 1 \right)}{dt}=\lambda_{2}(1)-\gamma I_{2}(1)-{(\phi(1)+\mu(1))I}_{2}(1)$$

$$\frac{{dI}_{2}\left( a \right)}{dt}={\phi\left( a-1 \right)I}_{2}\left( a-1 \right)+\lambda_{2}\left( a \right)-\gamma I_{2}\left( a \right)-{\left( \phi\left( a \right)+\mu\left( a \right) \right)I}_{2}\left( a \right)$$

$$\frac{{dR}_{2}\left( 1 \right)}{dt}=\gamma I_{2}\left( 1 \right)-\omega R_{2}(1)-{(\phi(1)+\mu(1))R}_{2}(1)$$

$$\frac{{dR}_{2}\left( a \right)}{dt}={\phi\left( a-1 \right)R}_{2}\left( a-1 \right)+\gamma I_{2}\left( a \right)-\omega R_{2}\left( a \right)-{\left( \phi\left( a \right)+\mu\left( a \right) \right)R}_{2}\left( a \right)$$

$$\frac{{dS}_{3}\left( 1 \right)}{dt}=\omega R_{2}(1)-\lambda_{3}(1)-(\phi(1)+\mu(1))S_{3}(1)$$

$$\frac{dS_{3}\left( a \right)}{dt}={\phi\left( a-1 \right)S}_{3}\left( a-1 \right)+\omega R_{3}(a)-\lambda_{3}(a)-({\phi(a)+\mu(a))S}_{3}(a)$$

$$\frac{dI_{3}\left( 1 \right)}{dt}=\lambda_{3}(1)-\gamma I_{3}(1)-{(\phi(1)+\mu(1))I}_{3}(1)$$

$$\frac{{dI}_{3}\left( a \right)}{dt}={\phi\left( a-1 \right)I}_{3}\left( a-1 \right)+\lambda_{3}\left( a \right)-\gamma I_{3}\left( a \right)-{\left( \phi\left( a \right)+\mu\left( a \right) \right)I}_{3}\left( a \right)$$

$$\frac{{dR}_{3}\left( 1 \right)}{dt}=\gamma I_{3}\left( 1 \right)-\omega R_{3}(1)-{(\phi(1)+\mu(1))R}_{3}(1)$$

$$\frac{{dR}_{3}\left( a \right)}{dt}={\phi\left( a-1 \right)R}_{3}\left( a-1 \right)+\gamma I_{3}\left( a \right)-\omega R_{3}\left( a \right)-{\left( \phi\left( a \right)+\mu\left( a \right) \right)R}_{3}\left( a \right)$$

$$\frac{{dS}_{4}\left( 1 \right)}{dt}=\omega R_{3}(1)-\lambda_{4}(1)-(\phi(1)+\mu(1))S_{4}(1)$$

$$\frac{{dS}_{4}\left( a \right)}{dt}={\phi\left( a-1 \right)S}_{4}\left( a-1 \right)+\omega R_{4}\left( a \right)-\lambda_{4}\left( a \right)-{\left( \phi\left( a \right)+\mu\left( a \right) \right)S}_{4}\left( a \right)$$

$$\frac{dI_{4}\left( 1 \right)}{dt}=\lambda_{4}(1)-\gamma I_{4}(1)-{(\phi(1)+\mu(1))I}_{4}(1)$$

$$\frac{{dI}_{4}\left( a \right)}{dt}={\phi\left( a-1 \right)I}_{4}\left( a-1 \right)+\lambda_{4}\left( a \right)-\gamma I_{4}\left( a \right)-{\left( \phi\left( a \right)+\mu\left( a \right) \right)I}_{4}\left( a \right)$$

$$\frac{{dR}_{4}\left( 1 \right)}{dt}=\gamma I_{4}\left( 1 \right)-{(\phi(1)+\mu(1))R}_{4}\left( 1 \right)$$

$$\frac{{dR}_{4}\left( a \right)}{dt}={\phi\left( a-1 \right)R}_{4}\left( a-1 \right)+\gamma I_{4}\left( a \right)-\mu\left( a \right)R_{4}\left( a \right)$$

The model has the following parameters;

- $b$ birth rate, which is updated to maintain a constant population size
- $\phi(a)$ age progression rate, which was calculated from the population demographic data from the UNDP [9]. A proportion of individuals are assumed to die (at an age-specific mortality rate $\mu(a)$) rather than progress, ie. $\phi\left( a+1 \right)=\phi\left( a \right)-\mu(a)$. The age fit of the age distribution of the model vs the data is shown in figure S1.
- $\gamma$ recovery rate from being infectious, where values used depended on the model ensemble
- $\omega$ heterologous waning immunity, where individuals were resistant to further dengue infection while recovered, but then progress to a new ‘susceptible’ stage (2-4).
- $\beta_{l}$ transmission coefficient for dengue associated with low viremia, corresponding to primary, tertiary and quaternary infection.
- $\beta_{h}$ transmission coefficient for dengue associated with high viremia. We assume that only secondary infection results in high viremia.

**

Figure S1. Comparison of the proportion of the population of ages 0-80 as reported within UN data (blue dots) and the modelled age-distribution within simulations (black dots) [9]

**

*Figure S2. Comparison of fitted model predicted age-specific seroprevalence and observed age specific prevalence as measured by survey data* [10,11]*. Comparable model-based estimates were made by averaging predicted mean incidence in the 50km around the area of the survey then extracting model-predicted age-specific seroprevalence for the specific value of incidence. Mean and range of estimates are shown in this figure with uncertainty due to different mathematical model parameterisations (Table S6 and S7). Blue points represent datapoints where the range of model predictions falls within the true value. The percentage of datapoints where the model prediction is within the true value are as follows: Age 1-4: 57%, Age 5-9: 80%, Age 10-14: 73%, Age 15-18: 63%.*

*Table S6 Summary of modelling assumptions here and comparison to the 8 models in Flasche et al., and implications.*

| *Model* | *Key structures* | *Assumptions on infectiousness* | *Duration of infectiousness (DOI - days)* | *Incubation period (days)* | *Assumptions on heterologous immunity* | *Implications for ensemble* |
| --- | --- | --- | --- | --- | --- | --- |
| *This study* | *4-stage ODE* | *All stages and disease outcomes transmit equally (unless Wolbachia infected)* | *Varied by ensemble*  *All stages and disease outcomes have same DOI* | *0 (not modelled)* | *Duration of waning immunity varied by ensemble* |  |
| *Duke* | *4-serotype ODE* | *All serotypes and stages transmit equally^1^* | *9*  *All serotypes and disease outcomes have same DOI^1^* | *0 (not modelled)* | *Not infectious if infected shortly after another infection. 2 years protection.* | *Ensemble should produce similar long-term outputs* |
| *Exeter/Oxford* | *4-serotype ABM* | *Secondary infections (independent of serotype) were up to x2 infective than others* | *4*  *All stages and disease outcomes have same DOI* | *2* | *Full immunity to strains previously encountered, and cross immunity for up to 1 year.* | *Ensemble may under-predict effectiveness as we do not account for increased transmission of secondary infection* |
| *Florida* | *4-serotype ABM* | *All serotypes and stages transmit equally* | *All serotypes have same DOI*  *Asymptomatic: 2*  *Mild case: 4*  *Severe case: 6* | *4.8* | *2 years serotype cross-immunity* | *Contribution of severe cases to transmission may be under-estimated but not used in model fitting* |
| *Imperial* | *4-serotype ODE* | *Symptomatic are x2 infectious as asymptomatic* | *4*  *All serotypes and disease outcomes have same DOI* |  | *1 year serotype cross-immunity* | *Ensemble may under-predict effectiveness as we do not account for increased transmission of secondary infection* |
| *Hopkins* | *4-serotype ODE* | *Symptomatic are x2 infectious as asymptomatic, but also varied by serotype?* |  |  | *1.1 year serotype cross-immunity* | *Ensemble may under-predict effectiveness as we do not account for increased transmission of secondary infection* |
| *Notre Dame* | *4-serotype ABM* | *All serotypes and stages transmit equally* | *4*  *All serotypes and disease outcomes have same DOI* | *6.5* | *2 years serotype cross-immunity* |  |
| *Sanofi* | *4-serotype ODE* | *Information not available* | *Information not available* | *Information not available* | *1 year serotype cross-immunity* | *Ensemble should produce similar long-term outputs* |
| *UWA* | *Stochastic ABM* | *All serotypes and stages transmit equally* | *6* | *16* | *0.5 year serotype cross-immunity* | *Ensemble should produce similar long-term outputs* |

*ODE – ordinary differential equation, ABM – agent based model*

*^1^The analysis in Nagao and Koella 2008 illustrated that increased transmissibility of individuals with antibody dependent enhancement (ADE) did not qualitatively change the observed disease dynamics.*

*Table S7 The eight different parameterisations of the mathematical model of dengue transmission*

| *Probabilities & parameters* | *Sanofi* | *Hopkins* | *Imperial* | *Duke* | *UF* | *UWA* | *Notre Dame* | *Exeter* |
| --- | --- | --- | --- | --- | --- | --- | --- | --- |
| Symptomatic - Primary | 20 | 53 | 45 | 34.2 | 45 | 30 | 30 | 32 |
| Symptomatic - Secondary | 36 | 100 | 85 | 71.4 | 60 | 60 | 60 | 38 |
| Symptomatic - Tertiary | 9 | 23 | 11 | 13.6 | 10 | 10 | 10 | 12 |
| Symptomatic - Quaternary | 9 | 0 | 11 | 13.6 | 10 | 10 | 10 | 12 |
| Hospitalisation - Primary | 11 | 0 | 1 | 5.3 | 10 | 11.1 | 11.1 | 3 |
| Hospitalisation - Secondary | 15 | 14 | 12 | 11.2 | 20 | 20.9 | 20.9 | 11 |
| Hospitalisation - Tertiary | 7 | 0 | 0.9 | 4 | 5 | 5.2 | 5.2 | 2 |
| Hospitalisation - Quaternary | 7 | 0 | 0.9 | 4 | 5 | 5.2 | 5.2 | 2 |
| Death | 0.05 | 0.03 | 0.05 | 0.078 | 0.078 | 0.078 | 0.078 | 0.084 |
| Duration of cross-serotype protection (days) (1/$\boldsymbol{\omega}$) | 365 | 365 | 365 | 730 | 730 | 730 | 730 | 730 |
| Duration of Infection (days) (1/$\boldsymbol{\gamma}$) | 4 | 4 | 4 | 9 | 4 | 5.5 | 4 | 4 |

*SI1.5. Estimating reduction in mosquito ability to transmit DENV using vector competence data*

In this section we recreate the relative vector competence curves for wMel *Wolbachia* infected *Ae. aegypti* presented in Carrington et al. 2018 [12]. Please see the original manuscript for all experimental details and model fit statistics.

$$logit(I_{m}) \sim\alpha V_{v}+{\beta W}_{m}+{\gamma B}_{v}+{\delta E}_{d}+{\varepsilon D}_{d}+\left( 1 | P_{p} \right)+\tau$$

Where:

$I_{m}$ = Presence of virus in the saliva (0-1)

$V_{v}$ = Virus serotype (DENV-1, DENV-2, DENV-3, DENV-4)

$W_{m}$ = Mosquito strain (Wild Type or wMel)

$B_{v}$ = DENV viremia

$E_{d}$ = Days after consuming infected blood meal

$D_{d}$ = Days after patient got infected

$P_{p}$ = Patient ID

$\alpha, \beta, \gamma, \delta, \varepsilon, \tau$ = parameters estimated by fitting the model

This gave the probability of a single mosquito becoming infectious in the days after biting an infectious human. To derive the probability of onward transmission, we take into account the probability the mosquito will survive through the Extrinsic Incubation Period (EIP) of the DENV. Mosquito survival was assumed exponential with a daily hazard of death of 0.1 [13]

Effectiveness was then calculated as follows:

Effectiveness of wMel = p(infectious bite from wMel *Wolbachia* mosquito on day x) = p(wMel mosquito survives to day x) * p(DENV has completed EIP in wMel mosquito by day x).

Where probability of completing EIP comes from the previous logistic model ($I$):

$$B_{W, x}=e^{-0.1x}I_{v,m=W,v,x,d,p}$$

And similarly for wild-type mosquitoes:

$$B_{WT, x}=e^{-0.1x}I_{v,m=WT,v,x,d,p}$$

Entomological effectiveness is then given by:

$${wMel}_{E}=\frac{\int_{x=0}^{x= \infty} B_{W, x}}{\int_{x=0}^{x= \infty} B_{WT, x}}$$

*Table S8 proportional reduction in transmission between wild type and Wolbachia infected mosquitoes*

| Virus Serotype | Low Viremia | | | High Viremia | | |
| --- | --- | --- | --- | --- | --- | --- |
|  | mean | low | high | mean | low | high |
| DENV1 | 0.558734 | 0.544821 | 0.577736 | 0.477116 | 0.46532 | 0.491765 |
| DENV2 | 0.573316 | 0.558117 | 0.597404 | 0.496545 | 0.479478 | 0.520478 |
| DENV3 | 0.566458 | 0.549527 | 0.593915 | 0.487187 | 0.467974 | 0.514731 |
| DENV4 | 0.603809 | 0.592821 | 0.619471 | 0.544714 | 0.532519 | 0.561047 |

Figure S3: Logistic regression model predictions for Wild Type (dashed) and wMel infected mosquitoes to be infected after imbedding a high viremia blood meal. Grey lines show 95% confidence intervals of model fit.


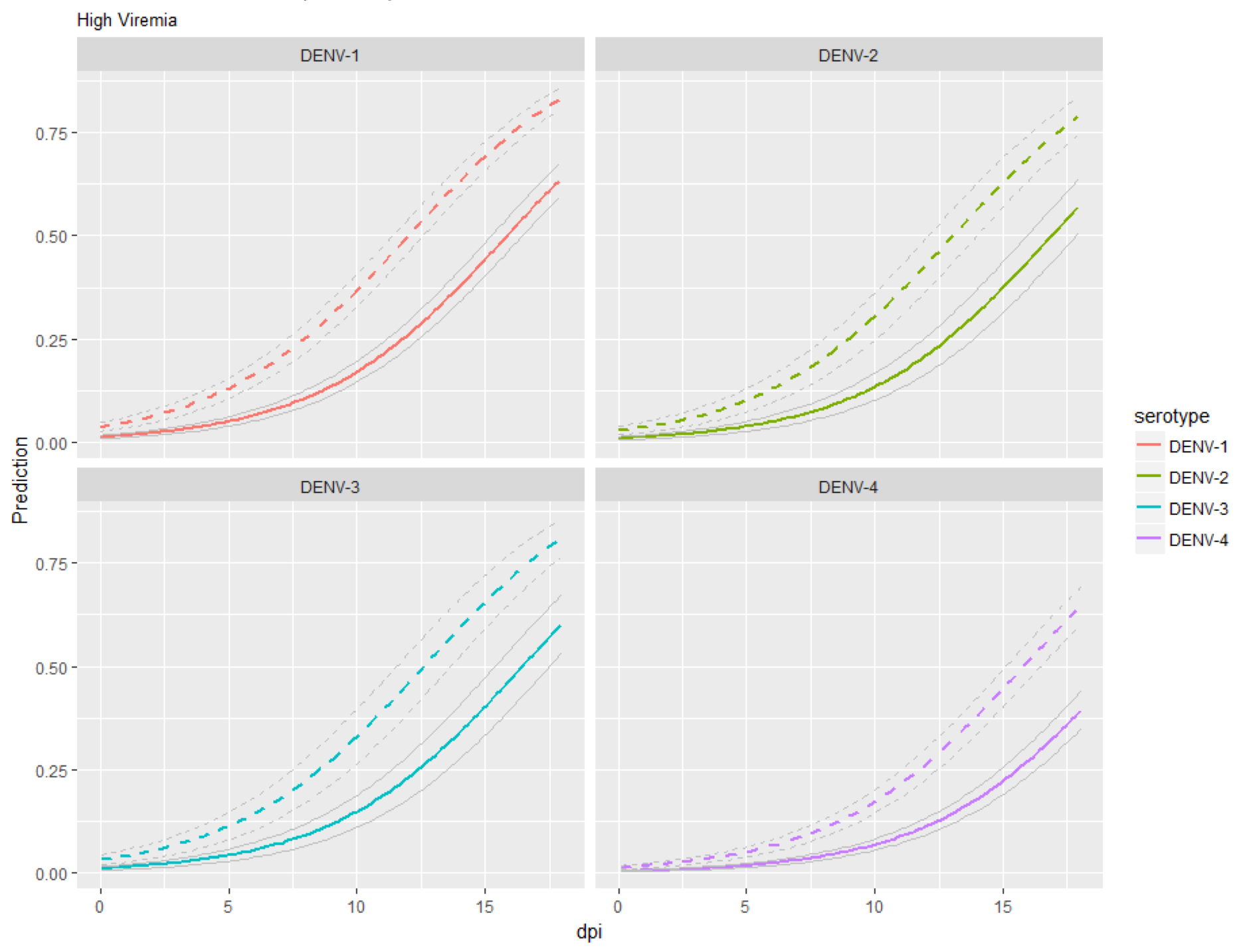


Figure S4: Logistic regression model predictions for Wild Type (dashed) and wMel infected mosquitoes to be infected after imbedding a low viremia blood meal. Grey lines show 95% confidence intervals of model fit.


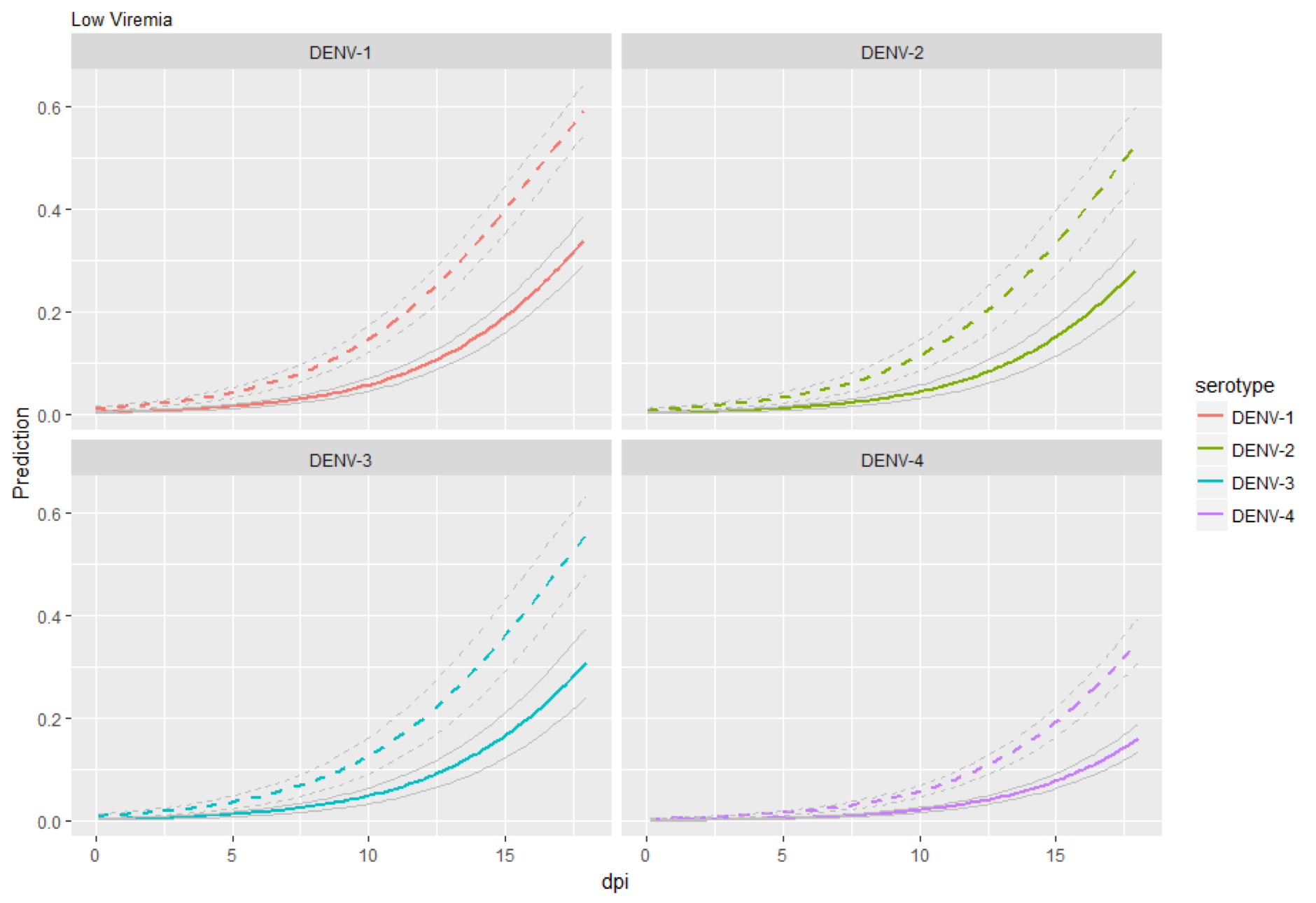


*Figure S5 Predictive performance of 100 occurrence data boosted regression tree models fit to national, regional and global datasets. Models are compared with regards to two metrics: proportion of land area with Multivariate Environmental Similarity Scores (MESS) greater than zero and Area Under the Curve (AUC) statistic using pair-wise distance sampling of the testing dataset. Perfect models would have scores of one for both metrics.*


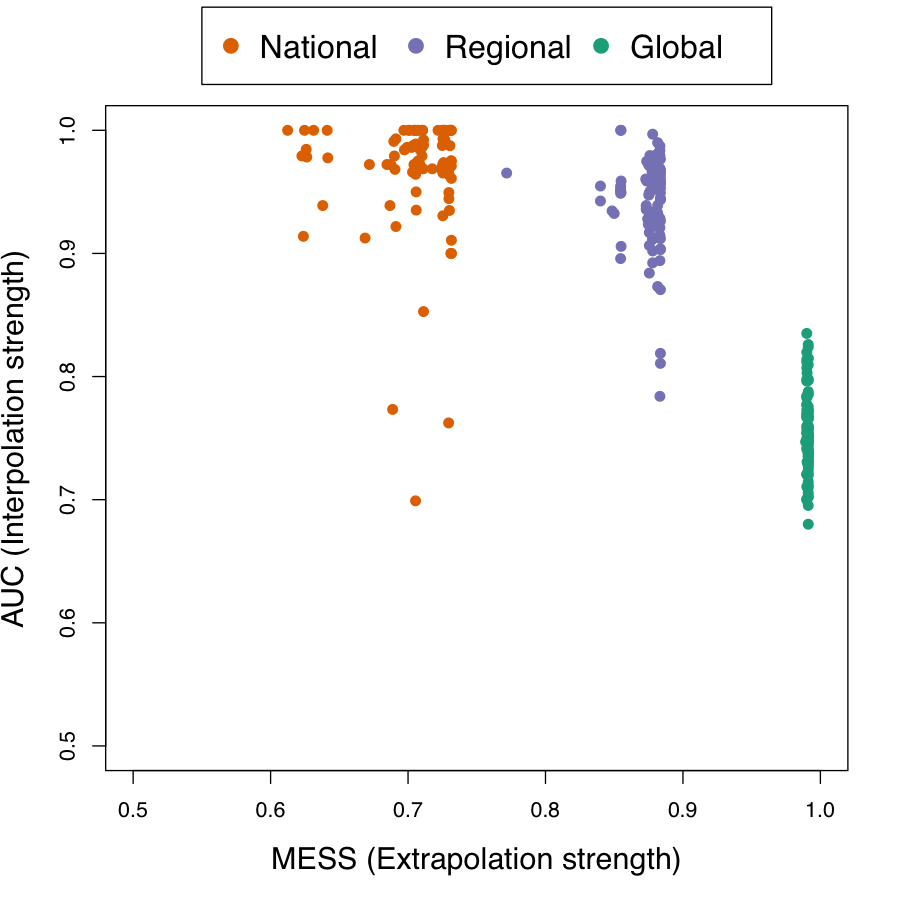


Table S9 Relative influence statistics for BRT occurrence mapping models at different scales

| Model scope | National model  (% contribution, 95% confidence intervals) | Regional model  (% contribution, 95% confidence intervals) | Global model  (% contribution, 95% confidence intervals) |
| --- | --- | --- | --- |
| Precipitation | 15 (12-18) | 26 (22-32) | 13 (12-16) |
| GDP per capita | 28 (23-34) | 20 (18-23) | 5 (5-5) |
| Urbanization | 21 (17-26) | 18 (16-20) | 5 (5-5) |
| Temperature suitability for dengue transmission | 17 (14-20) | 16 (13-18) | 68 (65-70) |
| Relative humidity | 12 (8-15) | 14 (10-16) | 6 (5-6) |
| Suitability for *Ae. albopictus* | 5 (2-9) | 4 (3-6) | 0 (0-1) |
| Suitability for *Ae. aegypti* | 2 (1-5) | 3 (2-4) | 2(2-3) |

Table S10 Comparative predictive performance of GBM and GLM-based models on a hold-out test dataset as measured by R-squared (log scale).

| Data type | R^2^ (95% confidence intervals) | |
| --- | --- | --- |
|  | GLM | GBM |
| Incidence data | 0.022 (0.0005 – 0.082) | 0.171 (0.068 – 0.309) |
| Seroprevalence data | 0.112 (0.0007 – 0.483) | 0.082 (0.00005 – 0.389) |

*Figure S6 Covariate effects plots of the regional BRT occurrence data mapping model. Y-axis shows the effect of each covariate on the response variable (dengue occurrence, higher = higher risk) and the X-axis shows values of the covariates. Precip2015 = minimum monthly precipitation in mm, RH2015 = minimum annual relative humidity, TSuit2015 = Temperature suitability Index for dengue transmission, aegypti2015 = suitability for Ae. aegypti, albopictus2015 = suitability for Ae. albopictus, GDP2015 = Gross Domestic Product in 2015 US Dollars. Urban15 = probability of land pixel being urban.*

*Figure S7 Fit of the Generalisaed Additive Models (GAMs) to the outputs of the Wolbachia effectiveness models. GAMs were fit with a monotonically decreasing constraint.*

**

*Table S11 Main strengths and weaknesses of occurrence, incidence and seroprevalence data*

| Type of data | Abundance (data points used in this analysis) | Representativeness | Bias | Spatial and temporal resolution |
| --- | --- | --- | --- | --- |
| Occurrence | High- 626 | Low – No clear denominator, opportunistic sample from research studies, reporting networks and news websites | High – potentially better coverage in areas better able to report digitally, increases in reporting over time, bias in site selection for research studies to high endemicity sites | High- 5 x 5km spatial resolution able to identify fine scale spatial heterogeneities in risk. Temporal distribution limited to annual due to time duplicate procedure to remove multiple reporting of cases from different reporting sources. |
| Incidence | Medium- 333 | Medium – Clear denominator (population in administrative areas), but numerator not always clear e.g. are private facilities always included, are outpatients always included, treatment seeking variations, etc | Medium – Theoretically national coverage, but biases towards areas with higher ability to diagnose and report dengue – typically highly populated areas with a regular dengue transmission e.g. Java. | Medium- High temporal resolution (up to monthly), but spatial resolution limited to second administrative unit |
| Seroprevalence | Low- 30 | High – clear experimental design that aims to gain a representative sample of the population although often age stratified to improve force of infection estimation. | Low- Bias addressed in experimental design. Sometimes minor biases introduced, e.g. challenges of sampling highly mobile individuals / populations that may be at higher risk. May also be bias in site selection – i.e. choosing areas based on historical dengue incidence | Low- Rarely done in > 10 sites nationally due to cost constraints. Typically, only able to measure long-term average incidence (over ~5-20 years) without the use of more complex methods, large sample sizes or paired samples (i.e. a serological cohort study). |

# References

1. Indonesian Bureau of Statistics. Indonesia - National Socioeconomic Survey 2014 (Combined) [Internet]. [cited 22 Jun 2018]. Available: https://microdata.bps.go.id/mikrodata/index.php/catalog/631

2. Nealon J, Taurel A-F, Capeding MR, Tran NH, Hadinegoro SR, Chotpitayasunondh T, et al. Symptomatic Dengue Disease in Five Southeast Asian Countries: Epidemiological Evidence from a Dengue Vaccine Trial. PLoS Negl Trop Dis. 2016;10: e0004918. doi:10.1371/journal.pntd.0004918

3. Elith J, Leathwick JR, Hastie T. A working guide to boosted regression trees. J Anim Ecol. John Wiley & Sons, Ltd (10.1111); 2008;77: 802–813. doi:10.1111/j.1365-2656.2008.01390.x

4. Wilson PD. Distance-based methods for the analysis of maps produced by species distribution models. Methods Ecol Evol. 2011;2: 623–633. doi:10.1111/j.2041-210X.2011.00115.x

5. Elith J, Kearney M, Phillips S. The art of modelling range-shifting species. Methods Ecol Evol. 2010;1: 330–342. doi:10.1111/j.2041-210X.2010.00036.x

6. Shearer FM, Huang Z, Weiss DJ, Wiebe A, Gibson HS, Battle KE, et al. Estimating Geographical Variation in the Risk of Zoonotic Plasmodium knowlesi Infection in Countries Eliminating Malaria. PLoS Negl Trop Dis. 2016;10. doi:10.1371/journal.pntd.0004915

7. R Core Team. R: A Language and Environment for Statistical Computing. Vienna, Austria: R Foundation for Statistical Computing; 2017.

8. Calcagno V, de Mazancourt C. glmulti: an R package for easy automated model selection with (generalized) linear models. J Stat Softw 12 (34), 1-29. 2010;

9. UNDP. World Population Prospects 2017. Geneva; 2017.

10. Sasmono RT, Taurel A-F, Prayitno A, Sitompul H, Yohan B, Hayati RF, et al. Dengue virus serotype distribution based on serological evidence in pediatric urban population in Indonesia. PLoS Negl Trop Dis. 2018;12: e0006616. doi:10.1371/journal.pntd.0006616

11. Prayitno A, Taurel A-F, Nealon J, Satari HI, Karyanti MR, Sekartini R, et al. Dengue seroprevalence and force of primary infection in a representative population of urban dwelling Indonesian children. Gürtler RE, editor. PLoS Negl Trop Dis. Public Library of Science; 2017;11: e0005621. doi:10.1371/journal.pntd.0005621

12. Carrington LB, Tran BCN, Le NTH, Luong TTH, Nguyen TT, Nguyen PT, et al. Field- and clinically derived estimates of Wolbachia-mediated blocking of dengue virus transmission potential in Aedes aegypti mosquitoes. Proc Natl Acad Sci U S A. 2018;115: 361–366. doi:10.1073/pnas.1715788115

13. Smith DL, Perkins TA, Reiner RC, Barker CM, Niu TC, Chaves LF, et al. Recasting the theory of mosquito-borne pathogen transmission dynamics and control. Trans R Soc Trop Med Hyg. 2014; doi:10.1093/trstmh/tru026
